# How to quantify information loss due to phase ambiguity in haplotype case-control studies

**DOI:** 10.1186/1471-2156-6-S1-S108

**Published:** 2005-12-30

**Authors:** Hae-Won Uh, Jeanine J Houwing-Duistermaat, Hein Putter, Hans C van Houwelingen

**Affiliations:** 1 Department of Medical Statistics and Bioinformatics, Leiden University Medical Center, Leiden, The Netherlands

## Abstract

Assigning haplotypes in a case-control study is a challenging problem. We proposed a method to quantify the information loss due to missing phase information. We determined which individuals were responsible for the information loss, and calculated how much information could be gained when the ambiguous individuals could be resolved by adding additional parental information.

## Background

Currently the majority of association studies using single-nucleotide polymorphism (SNP) markers for complex diseases are case-control disease-marker studies. In this paper, we consider a limited number of SNPs within a candidate region, with the aim of estimating haplotype frequencies and haplotype effects on disease status. This approach requires information about how to assign haplotypes from the observed genotypes. This *phase *information can be inferred using statistical procedures such as the expectation-maximization (EM) algorithm.

As Hodge et al. [1] showed, in general the probability of not being able to assign haplotypes with certainty increases with the number of the loci, and with the allele frequencies approaching 0.5. Accepting the "best" configuration of haplotypes as the "real" haplotype without critically examining data it might lead to misleading results. Therefore it might be useful to screen data beforehand using some measure of uncertainty.

There exists software with an option to print out all possible haplotype configurations with corresponding posterior probabilities. We wondered whether we could use this extra information to settle some of the current issues in haplotype analysis: how do you determine which individuals are responsible for the information loss, and how much information do we gain when parental genotypes were available?

With these issues in mind, we first defined the information loss as complete data information (without uncertainty) minus the observed information [2]. Under the assumption of Hardy-Weinberg equilibrium (HWE), we first considered the information content of each individual according to the diagonal elements of the information matrix. Considering the correlation between haplotypes, we employed D-optimality [3], which maximizes the determinant of the observed information matrix. With this measure, forward step-wise selection was applied to select the individuals that potentially yield the largest gain in information.

## Methods

Suppose we have a sample of *n *unrelated individuals from a population. From each individual we observe *m *multilocus SNP genotypes. Under HWE, the distribution of haplotypes is assumed to be multinomial, and the joint distribution of the paired haplotypes is equal to the product of the two marginal distributions. The haplotype will be described by a *k*(= 2^*m*^) dimensional vector *H *with its elements 0 or 1, and *P*(*H*_*i *_= 1) = π_*i *_denotes the frequency of haplotype *i *∈ {1, ..., *k*}. If there is no uncertainty, then for an (ordered) haplotype pairs (*H*_1_, *H*_2_) of one individual, *j *may be described with a *k*-vector *H*_*ind, j *_= *H*_1 _+ *H*_2_, where *H*_*ind, j *_∈ {0, 1, 2}, so-called haplotype dosage. Let *C *denote the covariance matrix of *H *as follows:



Using a natural parameterization of π:  where *α *= ln π, the total information with no phase ambiguity in the data is *I*_*tot *_= 2*nC*, and the covariance of estimated π is . In the case of uncertain haplotypes the total information from *n *individuals that is contained in the observed data is given by , where *L*_*i *_denotes the individual information loss due to phase uncertainty. As Louis [2] observed, this can be nicely interpreted as "*observed information = complete data information - missing information"*. Since we lose information, the covariance of estimates will increase: , approximately. So when we have no ambiguities in our data, *L*_*i *_= 0, and the covariance becomes simply *C*/2*n*.

We first investigated the diagonal elements of *L*_*i *_in cases. Although the use of the trace of *L*-matrix (A-optimality [3]) is an intuitive method to select individuals who need additional information, it does not consider the possible correlations of the parameters. Instead we propose to maximize the determinant of the information matrix based on D-optimality [3].

Finally, the real interest lies in quantifying the information loss due to haplotype ambiguities in the setting of case-control studies. This can be achieved by considering cases and controls separately as the two independent sample problem, and by combining the results using a (multiplicative) disease model: for example, by minimizing , where |·| denotes the determinant.

## Results

After performing a linkage analysis for the microsatellite markers, we analyzed SNP packet 153, including the microsatellite marker D03S0127 and 19 SNPs. Our example case-control data consist of 200 unrelated subjects and three loci. The case population consists of 100 affected offsprings selected from each family of Danacaa population replicate 8. To select a suitable subregion for our purpose we employed the sliding scores [4], and decided to study three-locus haplotypes based on B03T3056, B03T3057, and B03T3058. The computations were done with the programming language R [5].

We quantified the information loss per haplotype by A-optimality (Table 1). For the "rare" haplotype **212 **in cases, the information loss reaches almost 54% with respect to the situation of no uncertainty. Note that the relative information loss compared to the maximum information (%) can be interpreted as (1 - *R*^2^) × 100, where *R*^2 ^is the haplotype uncertainty measure by Stram et al. [6]. We can already detect different missing patterns between haplotypes.

The next question is: if you have options to solve the phase problem by collecting the additional family information, which individual would you select first? Using A-optimality we calculated the information loss per individual and per haplotype for cases in Table 2. We grouped individuals with identical genotypes, the order of the group identifications being determined by the trace of *L*_*i *_(the column "Tot. loss"). The characters of the group identifiers denote the genotypes at the SNPs, where **1 **and **2 **stand for homozygotes 1/1 and 2/2, respectively, and **H **denotes a heterozygote 1/2. The values of the last row give the information loss per haplotype as in Table 1. The highest label (**HHH**) denotes the group with highest loss, therefore potentially having the highest information gain. Hence, applying A-optimality the order of groups to be selected is: **HHH**, **H1H**, **HH1**, etc.

**Figure 1:**
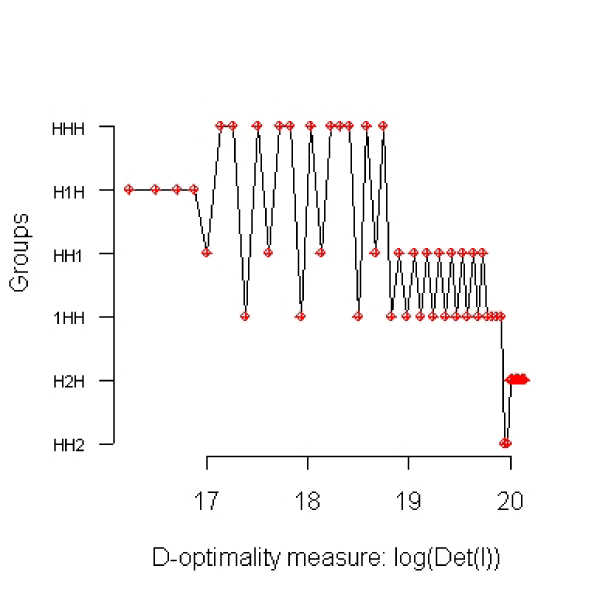
**Forward stepwise selection of the informative individuals based on D-optimality in 100 cases by maximizing |*I*_*tot*_|**. (1) The group identification denotes the genotypes at SNP: '1' and '2' represent homozygotes 1/1 and 2/2, 'H' a heterozygote 1/2. (2) The y-label is ordered by A-optimality (the highest 'HHH' group for the first selection, the 'H1H', 'HH1', etc), the red points by D-optimality. So the first individuals to be selected are 'H1H' group, not 'HHH', and hence it shows discrepancy using two different measures. The jumps between groups indicate the correlation between parameters.

Figure 1 shows the forward selection of individuals using the D-optimality criterion. The groups in the y-labels are ordered as in Table 2. Applying D-optimality we clearly see the potentially most informative persons are those with genotype **H1H**, and not the group of persons with three heterozygous loci, **HHH**. Hence, Figure 1 also illustrates the discrepancies in using two different criteria. Heuristically we might explain this as follows. In Table 2, the haplotype **111 **has the largest information loss. Within **111 **the individuals contributing the largest loss are the type **H1H**. Selecting (or resolving) one individual in this group will change the table, and we repeat the procedure. While Table 2 only represents the diagonal elements, Figure 1 gives a more complete representation of the structure of the loss matrix. Specifically, the jumps between the groups are caused by the correlations between the parameters. Moreover, at the beginning of the selection procedure we gain more information than at the end.

Observe that the above results are valid under the assumption that we could completely resolve the ambiguous haplotypes. When we actually added the parental information for this data, we could resolve about 71% of ambiguous individuals (number of cases = 100). Because it would depend heavily on the structure of data, for general usage we calculated the expected loss conditional on all possible parental genotypes. Using A-optimality, approximately 65% of information loss in average could be recovered.

## Conclusions and Discussions

The expected loss considering all possible (and compatible) parental genotypes does not differ much between the genotypic groups; it does not matter whether the individual is heterozygous on 2 loci, or 3 loci. For example, all heterozygous individuals might have two heterozygous parents (**HHH**), or two homozygous parents (father with type **111**, mother **222**). It clearly depends on the allele frequencies, hence on the structure of data. Our on-going investigation shows that the selection patterns also depend strongly on the questions asked; that is, whether we are interested in each group, in pooled groups, or in terms of haplotype risks in "minimizing error" or in "maximizing power".

Although selecting the informative individuals based on A-optimality is not as accurate as the method based on D-optimality, it is an intuitive method to understand the structure of uncertainty of the data. However, in some situations when the correlations of the parameters are not ignorable, our proposed methods might give more insight into the data. In our future work, we will investigate haplotype effects on disease status and some other extensions: focusing on "interesting" haplotypes, including missing data, or studying the behavior with an increasing number of SNPs.

## Abbreviations

EM: Expectation maximization

HWE: Hardy-Weinberg equilibrium

SNP: Single-nucleotide polymorphism

## Authors' contributions

H-WU performed the analyses and wrote the manuscript. H-WU and JJH-D carried out the preliminary linkage analyses. All authors participated in the development of the methods, interpreted of the results of the analysis, read the manuscript, and approved the final manuscript.

## Figures and Tables

**Table 1:** Information loss per haplotype based on the diagonal of information matrix in 100 cases, and *R*^2 ^measure.

Haplotype		cases			*R*^2^
		
nr	SNP-s	Loss	max^a^	%^b^	
1	111	9.51	37.18	25.57	0.7443
2	112	5.74	18.64	30.79	0.6921
3	121	8.11	43.28	18.74	0.8126
4	122	4.34	9.05	47.93	0.5206
5	211	4.49	10.11	44.39	0.5560
6	212	2.69	5.02	53.58	0.4641
7	221	5.35	16.07	33.32	0.6668
8	222	3.54	20.77	17.06	0.8293

^a ^max, the maximum information that is contained in a haplotype

^b^%, relative information loss compared to the maximum information.

**Table 2:** Loss of information in 100 cases per individual and per haplotype: '1' and '2' represent homozygotes 1/1 and 2/2, 'H' heterozygote1/2.

Group	Genotype	No.	111	112	121	122	211	212	221	222	Tot. Loss^a^
1	**HHH**	11	0.241	0.152	0.139	0.049	0.049	0.139	0.152	0.241	1.163
2	**H1H**	4	0.249	0.249			0.249	0.249			0.996
3	**HH1**	12	0.246		0.246		0.246		0.246		0.984
4	**1HH**	15	0.194	0.194	0.194	0.194					0.774
5	**H2H**	8			0.091	0.091			0.091	0.091	0.363
6	**HH2**	2		0.083		0.083		0.083		0.083	0.331
7	**2HH**	0					0.195	0.195	0.195	0.195	0.780
	OK	48									
	Total	100	9.506	5.739	8.113	4.337	4.490	2.690	5.354	3.545	43.77

^a^Tot. loss, _________________________.
